# “It's really hard to strike a balance”: The role of digital influencers in shaping youth mental health

**DOI:** 10.1177/20552076241288059

**Published:** 2024-10-18

**Authors:** Emily Adeane, Karolina Stasiak

**Affiliations:** 1 School of Psychology, 1415 University of Auckland, Auckland, New Zealand; 2 Department of Psychological Medicine, 1415 University of Auckland, Auckland, New Zealand

**Keywords:** Social media, adolescents medicine, mental health psychology, qualitative studies, health communications general

## Abstract

**Objective:**

Social media influencers have enormous sway with youth; however, little is known about how they communicate about mental health and how young people respond to this content.

**Methods:**

This study used semi-structured, remote interviews with 31 participants aged 16 to 24 years to explore how young people feel about the way social media influencers discuss mental health online. Thematic analysis and member-checking processes were used to identify five themes, each reflecting a tension in how participants viewed a specific aspect of influencer content.

**Results:**

Themes included the style (casual or serious), volume (inadequate or excessive), focus (distress or solutions), source (lived experience or professional advice), and sponsorship status (persuading or exploitative) of influencer content.

**Conclusion:**

Young people appear to hold widely varying, nuanced preferences regarding influencer mental health content. Notwithstanding some risks and limitations, influencers can generate engaging mental health content—their potential role in shaping youth mental health and other health communication efforts warrants further exploration.

## Introduction

Social media has transformed global connectivity, especially among the youth, making it a fundamental part of their everyday lives.^1^ Mental health is a critical issue for this demographic, with an increasing incidence of psychological challenges.^2,3^ While the internet's role in providing mental health support and facilitating peer interaction is well-established,^4^ the rise of social media influencers or simply ‘influencers’ introduces new dimensions to mental health communication.

Influencers are ‘micro-celebrities’ engaging vast audiences, from thousands to millions, through daily updates.^5^ They range from traditional celebrities to individuals who have gained fame online.^6^ Many operate across several sites like Facebook and Instagram, while others specialize in platform-specific content, such as lengthy ‘vlogs’ by ‘YouTubers’ or brief clips by ‘Tiktokkers’.^7^

### Impacts of influencers

Influencers hold significant sway, followed by nearly three-quarters of young Americans.^8^ Their perceived trustworthiness, relatability, and authenticity surpass traditional celebrities, cultivating intimate ‘parasocial’ relationships through frequent, candid posts and high audience engagement.^9–11^ The ubiquitous nature of social media and influencers’ ability to manipulate platform algorithms for increased visibility further amplifies their impact.^12,13^

As ‘opinion leaders’, influencers often advertise products via ‘sponsored content’ despite risks to audience engagement.^14,15^ Emerging health communication research indicates influencers can affect health knowledge and beliefs on topics like anti-smoking^16^ and vaccination^17^ and even health behavior, including diet, exercise, and adherence to public health measures.^18–20^ These ‘health influencers’ may also have diverse effects on psychological wellbeing. In a quantitative survey of over 1000 young adults, Campbell^21^ found that those who follow health influencers reported both higher wellbeing and greater distress compared to non-followers.

### Influencers and mental health

Most research linking influencers and mental health focuses on the psychological impacts of their content, such as upward social comparison leading to eating disorders.^22^ However, the study of influencer mental health content is relatively novel, consisting primarily of ethnographic case studies on individual YouTubers disclosing psychological conditions.^10,23–25^ Comparing four influencers, Lind and Wickström^26^ suggested influencers contribute to unrealistic and minimizing mental health discourses on self-management and self-love. Recent research also explores the phenomenon of mental health professionals becoming influencers, highlighting their educational potential alongside ethical concerns about credibility and confidentiality.^27,28^

While these content analyses offer insights into how influencers discuss mental health, such studies can only speculate about the potential effects on young audiences. Analysis of online comments sections suggests followers often find influencer mental health content helpful and relatable, enhancing peer support and help-seeking, though some consider it ‘triggering’.^23,29,30^ However, while comments offer real-world insights into audience responses, it is often not possible to discern commenters’ ages or nuanced views, highlighting a need for more in-depth methodologies such as interviews.

Digital interview studies on help-seeking suggest that youth view influencers as valuable information sources capable of encouraging help-seeking but hold concerns regarding ulterior, commercial motives.^31^ Similar concerns were raised in qualitative studies by Lehto^32^ and Thorn et al.,^33^ who reported audiences displayed mixed reactions to influencers promoting therapy services and their suitability in suicide awareness campaigns, respectively. However, such findings appear mainly incidental, with influencers not included in the central research question.

Few studies have intentionally explored audience perceptions of influencer mental health content. Koinig^34^ found that survey respondents appreciated influencers’ ‘human touch’ compared to non-influencer mental health content, suggesting their potential for health promotion. Qualitative exploration is further limited. A notable exception, Harris et al.^35^ reported that youth focus groups found ‘health YouTubers’ (including mental health creators) relatable and informative yet held concerns regarding the glamorization and generalization of mental health issues. These findings indicate diverse and nuanced perspectives, highlighting the need for broader research across platforms.

### Current study

Overall, there is a lack of in-depth, qualitative research which intentionally explores how young audiences perceive and engage with influencer mental health content from across the web. To fully grasp the dynamics of influencers’ mental health content, a comprehensive examination of its nature, the viewpoints of those engaged, and its potential effects on youth are essential. Therefore, we conducted two parallel qualitative studies, interviewing (a) young adults and (b) social media influencers. The paper at hand specifically reports on the findings from the first study, an exploratory investigation designed to uncover how young audiences perceive mental health content shared by influencers. We sought to understand what aspects of this content attract, engage and deter young people and how youth experience or even interact with such posts. The study also aimed to explore the rich nuances and tensions in the audience's perceptions and preferences regarding influencer mental health discourses and the broader implications of such content on followers’ own wellbeing, including their knowledge, beliefs and behaviors relating to mental health.

## Methodology

### Study design

This qualitative, exploratory research utilized in-depth interviews to investigate youth perceptions of influencer mental health content. Various remote interview formats (phone, videoconferencing, and online messenger) were employed to facilitate accurate communication of digital practices.^36^ Grounded in a youth empowerment approach and social constructionist epistemology, this study used reflexive thematic analysis to explore nuanced participant perspectives.^37–39^ As researchers and psychologists, we recognize potential differences in understanding mental health and wellbeing compared to participants. While the first author (EA) related to participants as a fellow young person and ‘digital native’,^40^ the co-author (KS) provided an outside perspective. The lead author's experiences as an online counsellor and prior investigations of digital youth practices inspired this study.^31^

### Recruitment

Researchers sought 25 to 30 participants via convenience, snowball, and purposive sampling. Participants were recruited nationwide through physical and digital ads across networks such as schools, youth organizations, social media groups, and universities. Eligibility criteria included being 16 to 25 years of age, an active social media user following at least one influencer, and being interested in mental health. Interested individuals contacted EA by email, phone, or social media (@nzinfluencerproj) to receive study details and arrange an interview. Prior to the interview, participants were asked if they had read and understood the participant information and consent forms, had no further questions and agreed to participate. Participants affirmative responses (e.g. “yes I consent”) were then documented in the interview transcripts as evidence of their consent. Ethical approval was obtained from the Auckland Health Research Ethics Committee (ref: AH22629), who approved this process of obtaining consent in lieu of a written form. Participants received NZ $30 (US $20) voucher and could opt for future acknowledgment.

### Cultural protocols

Health research in New Zealand should honor the rights and cultural practices of the indigenous Māori people guaranteed by Te Tiriti o Waitangi.^41^ We adapted Māori cultural practices commonly employed in research,^42^ including offering to open and close interviews with karakia (prayer) and waiata (song, via audiovisual link). The interviewer EA introduced herself using an abbreviated pepeha (introduction highlighting ancestral connections) and exchanged demographic details with participants as a means of whakawhanaungatanga (relationship building).

### Participants

Thirty-one young people took part in the study. See Table 1 for sample characteristics.

**Table 1.** Demographic characteristics of the sample.

	*n*	(%)
Participants	31	100%
Age (M/R)	20.71 years	16–24 years
Gender		
Female	21	68%
Male	9	29%
Nonbinary	1	3%
Ethnicity		
NZ European/Pākeha	16	52%
Asian	10	32%
Pacific Peoples	3	10%
Other European^a^	4	13%
Māori	2	6%
Black/African American	1	3%
Sexual orientation		
Heterosexual	26	84%
Bisexual	3	10%
Pansexual	1	3%
Did not disclose	1	3%
Location		
Auckland	23	74%
Other NZ cities	4	13%
Rural areas	4	13%
Occupation		
Student (secondary and tertiary)	19	61%
Health & Social Services	5	16%
Hospitality & Retail	4	13%
Business	4	13%
Agriculture	2	6%
Education	1	3%
Stay-at-home parent	1	3%

^a^ Including the United Kingdom and Slavic countries.

### Data collection

Interviews were conducted between February 2022 and January 2023, remotely from the researcher’s base in Auckland, New Zealand. Primary recruitment concluded after approximately 25 interviews. Additional, targeted recruitment continued for approximately 3 months to enhance the representation of Māori, Pacific and male participants and reach data saturation. The interviewer (EA) informed participants that this doctoral research was part of her clinical psychology training. Interviews were conducted via dedicated research phone on participants’ choice of platform (WhatsApp online messenger = 23; Zoom videoconference = 5; phone call = 3). We used a semi-structured interview guide to explore participants’ observations and opinions regarding influencer mental health content (see Table 2: Sample Interview Questions). Where practical, participants could share relevant images from their own or publicly available social media accounts to facilitate communication of digital practices. Most interviews lasted between 60 and 90 minutes. Two interviews lasted approximately 120 minutes due to delays in participant text responses. Following interviews, participants were invited to receive optional study updates and opportunities (including member-checking processes).

**Table 2.** Sample interview questions.

Area of inquiry	Sample questions
General perceptions and experiences	How would you describe a social media influencer? How many do you think you follow?
Experiences and perceptions of influencer mental health content	Do the influencers you follow talk about their mood or wellbeing? If so, how? Do you know any influencers who have been open about their mental health treatment? How do you feel about this?
Questions relating to the future potential of influencers	How could we use influencers to change how much young people know about mental health? How could we use influencers to help young people reach out for help?

### Data analysis

Researchers exported written and visual interview data into transcripts, with audio data transcribed by EA or a third party under a confidentiality agreement. Transcripts were then anonymized and securely stored on a university computer. Using NVivo software (version 1.7.1), EA conducted deductive, reflexive thematic analysis using Braun and Clarke's^43^ six-step method. This involved repeatedly reading transcripts and systematically generating initial codes based on short phrases such as ‘use of humor’. All codes were collated, and broad patterns or themes were identified, reviewed, labelled and visually mapped out. The analysis was then integrated into a coherent written narrative. Steps were revisited as needed, and a reflexive log was maintained to track analytical evolution.

Researchers collaborated and reached analytical decisions through consensus,^44^ supporting analytical claims with direct quotes (identified using pseudonyms). Quotes are presented exactly as received, preserving the authentic language, stylistic nuances, and textuality typical of instant messaging, including original capitalization, slang, abbreviations, and emojis. Spelling mistakes are corrected for readability, while maintaining the original essence to offer a genuine representation of young participants’ voices. Analysis adhered to the ‘COREQ’ checklist for comprehensive and transparent reporting.^45^ We also conducted multiple optional ‘member-checking’ processes to ensure data fidelity and uphold participant rights to tino rangatiratanga (sovereignty).^37^ Participants could review and amend their transcripts within 14 days, followed by a short interview summary. Participants could also complete a short online feedback form on the preliminary findings, receiving an additional NZ$30 voucher (US $20) as thanks.

## Findings

**Figure 1.**
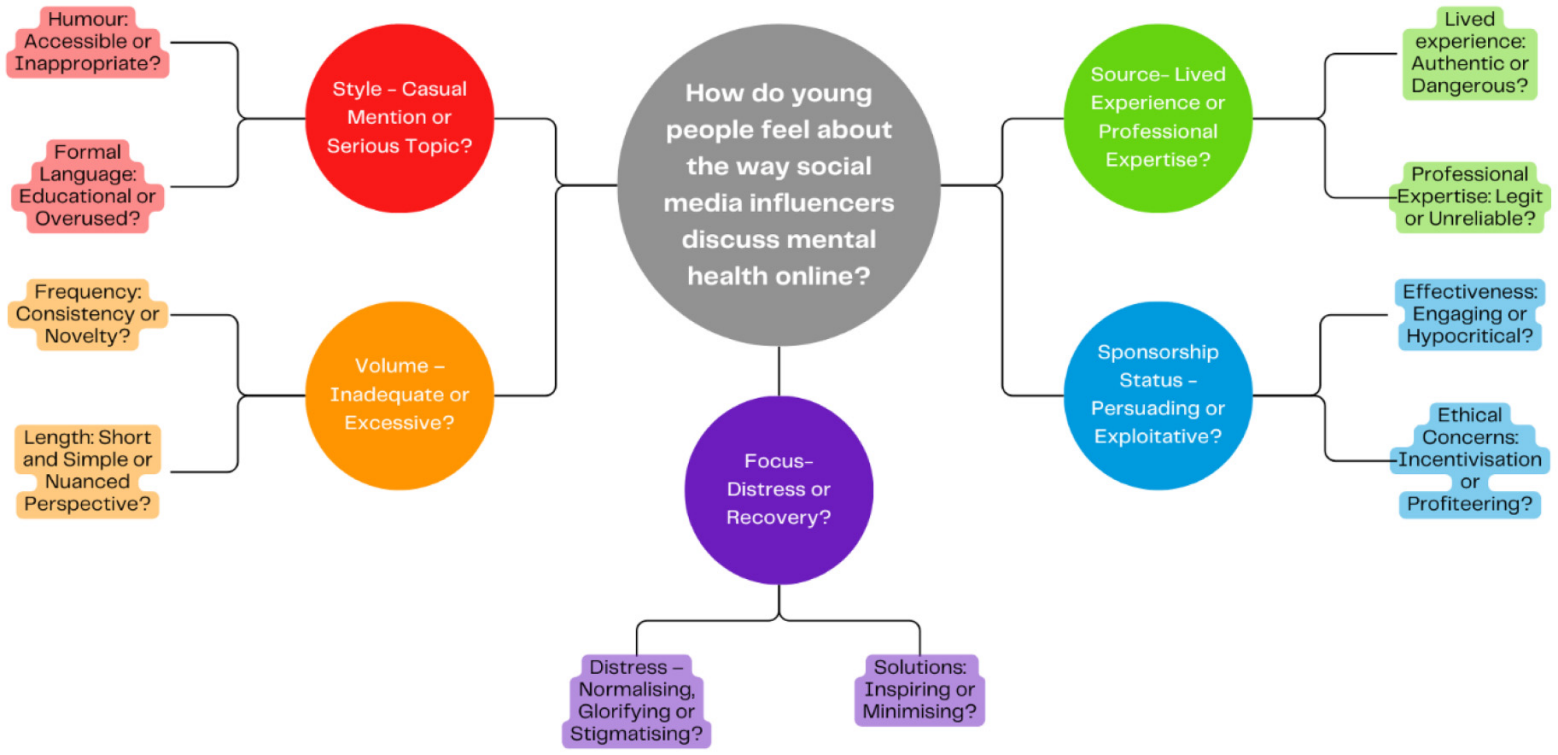
Theme map.

The analysis revealed several key tensions in participants’ perceptions of how influencers address mental health online. The findings were organized into five distinct themes, each exploring a tension within participants’ preferences and perceptions of a particular aspect of social media content. These included tensions regarding the style (casual or serious), volume (inadequate or excessive), focus (oriented towards distress or solutions), source (lived experience or professional advice), and sponsorship status (seen as either persuading or exploitative) of mental health content. These themes are further broken down into subthemes, as depicted in Figure 1.

### Theme 1: style—casual mention or serious topic?

Participants expressed varying perspectives regarding the style of influencer mental health content, debating the suitability of casual versus serious approaches, particularly the use of humor and formal language.

#### Humor: accessible or inappropriate?

The use of humor was contentious. Some championed serious approaches to mental health, arguing “it's not a joking matter” (Lori). However, participants like Jade believed influencers should not “shy away” from humor, noting it can make mental health discussions more accessible:

I find it takes the edge off a little bit in a good way. It helps him to touch on important discussions without being too heavy about it. I also find it takes away the taboo of it to openly make jokes.

Others found humor offensive, suggesting it trivialized sensitive issues. They criticized how “young influencers joke about their depressions/crippling anxiety to be relatable, which I think makes light of these potentially really harmful issues, and makes it seem like it's something everyone has and should just deal with” (Elizabeth). Influencers who consistently use humor may be less likely to offend audiences, as Jade observed, “I haven’t personally seen any people having negative reactions […] Probably because he's known for his slightly darker sense of humor.” Joking about shared challenges can also serve as a “pick-me-up” (Lori) or subversively “make depression ironic” (Peyton). However, Peyton also warned against overuse, particularly of memes or internet in-jokes, as “using memes for MH [mental health] has become uncool […] it's just not funny now.”

#### Formal language: educational or overused?

Influencers’ use of clinical terminology was similarly divisive. Some suggested that clinical terms can help increase mental health awareness and reduce stigma. For instance, Summer “learned more about borderline personality disorder through an influencer, which helped me to understand someone who I am close with as they have bpd.” Disclosing diagnoses also makes influencers identifiable role models, as Nathan explained, “like she had ADHD [too], you know, like so inspiring for me.”

However, there were significant concerns regarding influencers’ ability to use clinical terms correctly, saying “words can get thrown down like I’m depressed or I’m anxious […] But they might actually just be sad, or they might just be going through a stressful time” (Wendy). Participants feared such generalizations may exacerbate issues with inaccurate self-diagnosis because “if you saw someone say like oh, this is, the symptoms of bipolar type two […] Most people would be like, ‘oh maybe I have that’” (Wendy). Participants criticized influencers perceived as using clinical terms to appear “trendy” (Holly), alongside those “using mental health terms as insults or quirks (e.g. ‘I’m so OCD!’)” (Jade), which could exacerbate stigma.

### Theme 2: volume—inadequate or excessive?

Participants expressed diverse views on the ideal volume of influencer mental health content, including the optimal frequency and length of posts. While many welcomed any discussion of mental health, others favored a ‘less is more’ approach.

#### Frequency: consistency or novelty?

Most participants preferred influencers to discuss mental health more often, noting “it does take the stigma away sort of thing if it's more talked about” (Wendy). Frequent discussions may make mental health appear more accessible and increase trust as when “they discuss it often […] their audiences feel they are chatting to them as a friend” (Hazel). Regularly posting about mental health was deemed critical for demonstrating genuine interest and authenticity, particularly when making recommendations, as when they “consistently talk about it […] it would give more credibility to the resource” (Linda).

Conversely, others criticized influencers who talked “excessively” (Nathan) about mental health, describing it as irritating “preaching” (Pavel) or “jumping on the train” (Nathan) of yet another cause. Others expressed frustration with how often influencers discussed personal struggles, hyperbolically suggesting “there's often a billion stories about them being upset, seems a bit whiny” (Steph). Summer warned this might overwhelm viewers, as “if the influencer is constantly talking about mental health and anxiety/depression it can be exhausting for a viewer to be constantly consuming this information and not actually helping with their wellbeing.”

Less frequent mental health content or recommendations may be more attention-grabbing “because it would be rare for them to promote it, I would be intrigued, I’d be like, ‘wow, this must be cool or like pretty interesting if they’re using it’” (Nathan). This surprise and novelty may make posts more compelling, as Hineatua explains of one influencer, “Due to him always being cheerful in his videos, I related more when he spoke out and I took his suggestions seriously.” Certain groups who discuss mental health less frequently may appear more authentic, as “when you hear it from a man who doesn’t talk about it as often, it kind of has more of an effect” (Nathan).

However, infrequent mentions can also appear odd or irrelevant, as Seb explains, “It would be very strange for the person that I follow to randomly have like a minute or two of their videos dedicated to talking about their mental health for their followers.” Such posts may also appear insincere or “contradictory” as “often influencers promote things which could be damaging to their followers’ mental health” (Linda). Several participants, mostly male, expressed disappointed that most influencers they follow “aren’t consistently posting about mental health” (Israel). They argued infrequent posts maintain the silence and stigma surrounding mental health, saying “all male influencers I have followed haven’t touched on this topic at all further repressing it” (Gabriel).

#### Length: short and simple or nuanced perspective?

Participants debated the optimal length for mental health content, contrasting preferences for concise, engaging content versus longer, more thorough explorations. Observing the rising popularity of short-form content and how “younger people are gravitating towards TikTok etc.” (Elizabeth), participants recommended influencers “keep it short and simple” (Anna) when discussing mental health. They argued that young peoples “attention spans are shorter” (Hineatua) and “just expect the answers to come immediately” (Pavel). Participants, therefore, praised “short and snappy tips that get straight to the point means you can quickly filter through irrelevant ones” (Willow).

Others suggested that longer content appears “more professional and the influencers seem more well, trustworthy” (Kaleb). Elizabeth argued that shorter formats “over-simplified” mental health, preferring “long-form content like YouTube, since they encourage giving a more nuanced perspective than 6-second clips on TikTok or attractive photos on Instagram.” Longer content, therefore, “helps you understand more” (Solomon), possibly because it is consumed more intentionally, whereas shorter content is “usually on the way, waiting for something. But then YouTube you sit down and then open your laptop” (Hope).

### Theme 3: focus—distress or solutions?

Participants identified two types of content. Distress-focused content showcases mental health challenges, including influencers crying or discussing grief, and was perceived as validating, glorifying or stigmatizing. Solution-focused content, such as wellbeing tips or recovery stories, was viewed as either inspiring or minimizing.

#### Distress—normalizing, glorifying or stigmatizing?

Perspectives varied on whether influencers who openly display psychological distress normalize, glorify or stigmatize mental health problems. Many commented that such displays, from crying to panic attacks, help demonstrate that distress is “completely normal” (Faith) and that mental health problems exist “on a spectrum just like everything else” (Hazel). Participants like Gabriel criticized influencers who “just disappear” during challenging times for reinforcing the unrealistic expectation that “you’ve got to be happy all the time.” Sharing distress regarding “collective issues” (Pavel), such as the Covid-19 pandemic, was particularly valued. Such content conveys “it's okay if you’re not feeling great about it too” (Wendy) and fosters connections so “it feels like everyone's going through something together” (Hazel). Displaying distress may also help youth with similar problems feel less abnormal; as Sara notes of one influencer, “by sharing her struggles, she shows people they aren’t alone.” Such posts may also prompt help-seeking as “when we see that the people were idolizing can struggle, it makes us feel like it's okay that we do too. If they have the courage to speak about it and acknowledge it, we can too.” (Priya).

Others feared displays of distress might unintentionally glorify mental health issues, presenting them as desirable, “like depression and anxiety have been trendy before” (Steph). When considering how “young people tend to model their behavior on them” (Connor), influencers may, therefore, prompt young people to reproduce ineffective coping strategies:

If you had an influencer who, you know, gone through all these emotional mental health struggles. And their solution to it was, ‘oh, you know, just, if you’re gonna self-harm, make sure you do it in a really safe manner’ […] it feels kind of counterproductive. You shouldn’t be advertising that kind of thing. (Seb)

Participants also expressed concerns that displays of distress attract “a lot of support and people offering their DMs” (Anna). Participants warned that increased engagement could inadvertently reinforce ineffective coping strategies. For instance, Elizabeth observed that an influencer’s binge-eating posts “got heaps of views. So in that way, she was kind of encouraged to keep going with this.” This interaction may escalate the influencers’ difficulties “because instead of people telling these large creators ‘bro, maybe you need help’ or NOT WATCHING, they watch the train wreck get worse and worse” (Peyton). Audience engagement might even deter influencers from help-seeking, as influencers are incentivized to “express hardship and don’t receive help because it helps them grow.” (Peyton).

Others suggested that distress-focused content may foster irritation and skepticism, ultimately increasing stigma. While many viewed distressed influencers as “much more ‘real’ “ (Priya) and authentic, others felt “skeptical” as “most people don’t solve their problems by broadcasting it in that way, so it seems a bit artificial to get attention” (Steph). They suspected influencers may be “utilizing mental chaos for views” (Peyton) or eliciting sympathy with “crocodile tears […] just doing it so that they can recover some positive reputation they may have had.” (Connor). Consequently, many felt unmoved or even irritated by displays of distress, describing them as “narcissistic” (Connor) or “whiny” (Steph). Participants appeared particularly critical towards influencers perceived as unproductively “wallowing in their feelings” (Peyton), leaving participants exasperatedly exclaiming “oH mY gOd jUsT bUy a ThErApIsT!!!” (Peyton). This may leave young people sensitive and skeptical of others expressing distress, perpetuating stigma as it “gives a bad impression for the people who actually do really suffer” (Steph).

#### Solutions: inspiring or minimizing?

Participants discussed whether influencers focusing on recovery and solutions inspire or minimize youth with mental health struggles. Participants confirmed that they “do see some stuff from influencers that has inspired me to try help with my own mental health” (Tausa’Afia). This may include encouraging youth to practice “skills and tools” (Katie). For instance, Anna “definitely started journaling and tried meditating based on an influencer raving about it.” Summer explained how she “seeks out inspiration from influencers on what to do when I’m feeling down” because they provide a reliable catalogue of ideas and “because it's more personal and these are people that I already look up to so their advice is valuable to me.”

Influencers may also provide hope, demonstrating the efficacy of treatments or self-help, with messages like “therapy actually helps really fast” (Solomon) or “you can significantly improve your mental wellbeing” (Hazel) through lifestyle changes. Others appreciated influencers’ realistic, nuanced depictions of recovery; for example, Elizabeth observed commenters “say she's really helped them with their anorexia, since she showed it's OK to gain weight and not have such tight control.” Brianna highlighted the popularity of such role models, explaining, “There is a large community of recovery/mental health (true mental health focused) accounts available.”

Conversely, some argued that the “hyper positivity” (Matthew) inherent in recovery content causes influencers to ignore or downplay their difficulties, potentially over-normalizing them and invalidating youth with similar experiences, with statements like “Oh I got checked into a MH ward but don’t worry about me don’t worry about it” (Peyton). Influencers may also be “overly optimistic” (Peyton) about recovery, minimizing the difficulties involved, alongside differences in individuals” capabilities, systemic factors, and privilege:

It can still be like, ‘oh just like spend $200 a week on therapy’. It's like, yeah, but like what if you really needed that $200 on like being able to get to work or like you need your like basic like needs met first. (Wendy)

Participants like Elizabeth also criticized influencers oversimplifying solutions, “encouraging people to do simple fixes which helped them” which, if unsuccessful cause demoralized youth to “beat themselves up because [they think] ‘why can’t I overcome this like this influencer can?’.”

### Theme 4: source—lived experience or professional expertise?

Participants expressed varying preferences for which influencers discuss mental health, distinguishing between mental health professionals or individuals with lived experience of mental health challenges.

#### Lived experience: authentic or dangerous?

Participants generally favored influencers with lived experience, as “it feels more authentic, if you […] went through it yourself” (Seb). Some viewed lived experience as merely advantageous, saying, “I don’t think you have to, but I do think that it would help” (Pavel), while for others, it was non-negotiable, commenting it “doesn’t come across well if it's something they’ve not personally experienced” (Priya). A lack of lived experience may appear insincere as “when an influencer is preaching without practicing it also has the risk of looking a bit performative” (Priya). Conversely, personal experience offers unique, compelling insights, such as a “real person's perspective as opposed to a trained doctors opinion” (Hazel).

However, participants also expressed concerns about lived experience, including that “listeners will rely on these people instead of seeking out professional advice” (Elizabeth). They argued that such influencers lacked sufficient experience to discuss mental health safely, commenting, “I don’t think unqualified influencers should be attempting to give out what should be professional advice/help for serious mental illnesses” (Brianna). As Wendy elaborated, when “they’ve not really got like an accreditation or the education […] it can actually be quite dangerous.” For instance, Seb outlines how influencers may promote ineffective means of dealing with distress such as self-harm, whereas professionals “have better methods to approach these things than someone who has simply experienced it.” Sharing lived experience may also distress followers like Nathan, who commented, “Obviously, whenever I see that stuff, it upsets me.” Influencers may also discourage help-seeking, either unintentionally, for instance, “discussing awful side effects […] could either influence someone to try medication or put them off the idea completely” (Hazel) or deliberately, “saying that psychiatry [is] just so kinda bogus” (Connor).

#### Professional expertise: legit or unreliable?

Most participants valued professional expertise, making content more credible and compelling as “I know that he's legit […] so, hearing him saying things at least makes me think that, hey, this might actually be something to look into” (Pavel). Influencer content featuring mental health professionals may enhance awareness and beliefs regarding treatment efficacy. For instance, Summer noted on a post featuring an influencers recorded therapy session, “There were a lot of people saying that the video helped them to realize that seeing a therapist […] can really help you with what you’re struggling with.” Such content may also motivate help-seeking by increasing young people's familiarity and trust with professionals:

He's the whole reason I started going to therapy. Yeah, he just made it okay and I was like if I could get a therapist like this, I think I could do a lot of good for myself. And I’ve heard the same thing from other people. (Pavel)

However, qualifications can be faked or misrepresented, as Kaleb wryly observed: “a lot of TikTok ‘doctors’ show their certificates to show that they are trustworthy.” Qualifications, therefore, do not ensure quality advice, as “just ’cause someone's got a doctor's name doesn’t actually mean that that is a reliable source of information” (Wendy). Furthermore, ethical and legal requirements regarding confidentiality and safety may limit professionals” ability to create engaging content. For instance, a psychiatrist must be “really careful about how he comes across” (Pavel) to avoid blurring the lines between medical advice and personal opinion.

While many participants recognized that lived experience and professional expertise were not mutually exclusive, some expressed clear preferences, saying, “If you haven’t had the experience, then at least have the qualification” (Seb). However, others like Wendy warned that “you need both” sources, as otherwise the discourse may be “too restricted that like you’re not finding useful information out.” Therefore, the “ideal” influencer may be “someone that's like educated in the field, but then also has first-hand personal experience” (Wendy). Some like Seb therefore suggested governments and educational institutions should “encourage or sponsor these influencers […] to be qualified in their field” potentially through creating programs like “a degree in being an influencer that focuses [on] mental health.”

### Theme 5: sponsorship status—persuading or exploitative?

Participants also debated the effectiveness and ethical considerations of paying influencers to create ‘sponsored content’, promoting mental health-related products and services, such as apps or counselling sites.

#### Effectiveness: engaging or hypocritical?

Participants generally viewed sponsored mental health content as an effective tool for spreading awareness and reducing help-seeking barriers, by getting “rid of any negative stigma regarding any of these services” (Chloe). They indicated a willingness to follow sponsored recommendations because “because I can relate to the influencer and it's like the influencer is incorporated into my daily life” (Hope). However, some criticized sponsored content as insincere, arguing “they’re only talking about it to get the sponsorship and not because, you know, they genuinely care about like their own mental health, and the health of their followers” (Seb). Skeptical participants, therefore, doubted the quality of advertised products because “obviously they’re being paid to promote it rather than just promoting something that they’ve found useful and beneficial” (Linda). To increase authenticity, participants suggested influencers share first-hand experiences of products and services, as “having open and honest and relatable unsponsored content helps build trust” (Grace). They also cautioned restraint in the frequency and number of sponsorships, as “if they’re promoting a different product or app or thing every day, I would be like what's going on here, you know?” (Nathan).

Influencers perceived as previously promoting pseudoscientific or emotionally damaging practices, such as extreme weight-loss tools, may also be viewed as hypocritical. As Linda suggests, “it could put people off engaging with the resources because it has come from someone who has possibly played a part in social media being detrimental to one's mental health.” However, she acknowledged that some repentant influencers may wish to “share more resources to negate this effect.” Such influencers may, therefore, be ideal advocates as “this way, at least the target audience gets the best exposure possible” (Brianna).

#### Ethical concerns: incentivization or profiteering?

Some argued it was inappropriate to profit from promoting mental health, saying “mental health is a big conversation, [it] shouldn’t be used as a marketing scheme” (Anna), or even immoral, as influencers should “just do it because it's the right thing to do” (Nathan). However, compensation appeared less offensive if it did not financially burden audiences:

I don’t think they should try make money out of people who are trying to help their mental health. Doesn’t seem super ethical. Unless it's quite cheap. But if the paid promo is for a free app well awesome! (Kaleb)

Some described influencers as exploiting mental health to sell other products, commenting, “there's people who can’t let a good crisis go to waste” and “use mental health to sell their latest new program, or exercise regime, or product” (Brianna). Such creators were characterized as misleading predators, or “people who prey on people's insecurities/mental health struggles to promote a product, like ‘Do this/buy my program and you’ll be rich/ripped/skinny and then you’ll be happy’” (Elizabeth).

However, many participants acknowledged the necessity of sponsored content, accepting it as “no different to any kind of advertising” (Brianna). Others believed it was entirely appropriate as “it's kind of what an influencers job is, just to sell and promote things” (Wendy) or praised it as advocacy, suggesting “it comes from a passion, and then they just happen to get paid for it” (Pavel).

## Discussion

### Principal findings

This study is among the first to qualitatively explore young people's perceptions of social media influencers’ mental health posts. Rather than providing a singular answer, this research adopts a dialectical approach, highlighting the diversity and contrast in opinions. Our findings reveal nuanced, varied and sometimes contradictory preferences about the style, volume, focus, source, and monetization of influencer mental health content. Casual and serious styles were compared, with humor viewed as relatable or inappropriate and formal language as educational or overused. The ideal frequency and length of mental health content also varied. Both high and low-frequency posts risked being perceived as inauthentic or dull, and participants debated the relative merits of shorter, engaging content or longer, nuanced material. Opinions also diverged on whether depicting distress normalized, glorified, or stigmatized mental health issues and if solutions-focused content was motivating or trivializing. The credibility and effectiveness of mental health professionals versus those with lived experience were also contested, and views on ‘sponsored’ content ranged from persuasive to exploitative.

### Comparisons to other research

Interest in influencer mental health content has recently surged, with several analyses published since this study's inception.^26,27,29^ Yet, few have qualitatively examined youth perceptions of this content, aside from Harris et al.^35^ and Campbell,^21^ who explored ‘health influencers’ broadly. Building on our prior work implicating influencers in what online content facilitates youth help-seeking,^31^ this study is among the first to broadly examine youth views on the full range and potential of influencer mental health content.

Preferences regarding the relative seriousness of content varied, supporting prior findings that some engage more with content treating mental health casually.^31,35^ While humor may enhance engagement,^31,46,47^ it may also offend, particularly regarding sensitive topics like suicide.^48^

Participants generally echoed calls for an increased volume of influencer mental health content, underscoring the importance of consistency in normalizing mental health and establishing trust and perceived expertise.^31,35,49^ Concerns regarding excessive posting have received less scholarly attention; however, the idea that influencers who discuss mental health less frequently are more noticeable and compelling is perhaps unsurprising given the rarity of this discourse among specific communities, such as young men.^50^

Participants reflected previous suggestions that displays of distress may either destigmatize or glorify mental health difficulties, potentially increasing inaccurate self-diagnosis or self-harm.^26,31,35^ While ‘solutions-focused’ content may be inspiring and educational,^21,27^ this study repeats warnings that influencers can oversimplify treatments and perpetuate unrealistic ideals of constant positivity and productiveness.^26,35^

This study supports findings that youth value the authenticity, relatability and practical insights offered by individuals with lived experience.^31^ However, concerns regarding the accuracy of influencers without formal training may be well-founded.^51,52^ This study also contributes to growing research on mental health professionals as influencers, exploring their potential benefits and ethical considerations.^27,28,31^

Despite the increasing prevalence of sponsored content,^53^ it remains controversial. This study supports the potential effectiveness of influencers in mental health promotion,^27,31^ while recognizing that youth responses may vary. While some express disapproval or indifference, many appear capable of accepting its necessity while maintaining concerns about its appropriateness.^15,35,54^

### Strengths and limitations

This is a robust qualitative study with a relatively large number of participants and supported by a concurrent study with social media influencers (reported separately). To the best of our knowledge, it is the first study of this kind in the mental health field. Unlike prior research, which primarily focused on examining influencer mental health content directly,^23,26^ this study provides a novel perspective by delving into young people's perceptions of such content, using in-depth interviews to generate rich insights. Furthermore, the study adopted a youth empowerment approach^39^ by offering participants a range of interview mediums, ensuring comfortable communication and enhancing data quality.^36^ Additionally, rigorous member-checking procedures enhanced data fidelity and upheld participants’ rights to data sovereignty.^37^

While this study provides valuable insights, several limitations should be considered. The generalizability of the findings is limited due to sample size, inherent subjectivity involved in qualitative research, and the potential self-selection bias of participants interested in mental health and influencers. Despite attempts to ensure greater diversity, the sample was weighted towards white, educated, female young people, with many above 20 years old. As a result, it is not possible to ascertain the prevalence of these views among wider audiences or draw definitive conclusions regarding the acceptability of influencers for most young people or the casual impacts of their online discussions. Larger-scale quantitative research, including the use of validated questionnaires, may offer a more comprehensive understanding of these matters, complementing the insights gained from this study.

## Conclusions

Our study revealed the multifaceted nature of young people's perspectives and the complexity of considerations and debates surrounding various aspects of mental health content disseminated by influencer. The breadth and complexity of perspectives underscores the absence of a one-size-fits-all approach, making it evident that what resonates with one individual may vastly differ from another. Therefore, influencers, mental health promotion agencies and others attempting to engage youth online may wish to consider adopting a flexible approach to cater to the diverse preferences of their audience- for instance, offering a blend of casual and serious styles, shorter and longer posts, and organic and sponsored content. No single influencer or account can cater to all preferences or provide every type of content. Fortunately, the internet provides a plethora of voices and avenues for discussing mental health, allowing young people to discover accounts that align with their interests and needs. Therefore, this study highlights the importance of having a broad spectrum of influencer mental health content available to young people, varying in style, volume, focus, sources, and sponsorship status. Additionally, this study highlights the crucial need for careful deliberation on incorporating lived experiences, professional insights, and sponsored content within influencer-led mental health discussions. It emphasizes the significance of maintaining authenticity in mental health conversations while ensuring a responsible depiction of challenges and their solutions.

Social media and its influencers are here to stay, and with young people's increasing interest in mental health, what influencers have to say on the topic is likely to carry increasing significance. While some approaches prove more effective and engaging than others, and there are some risks and limitations, influencers exhibit a remarkable ability to create mental health content that resonates with youthful audiences. Consequently, mental health researchers and professionals should devote greater attention to influencer-driven mental health content, considering their potential role in promoting mental health campaigns and services.
